# Binary THz modulator based on silicon Schottky-metasurface

**DOI:** 10.1038/s41598-022-23534-w

**Published:** 2022-11-07

**Authors:** Saeedeh Ahadi, Mohammad Neshat, Mohammad Kazem Moravvej-Farshi

**Affiliations:** 1 Nano Plasmo-Photonic Research Group, Faculty of Electrical and Computer Engineering, Tarbiat Modares University, P. O. Box 14115-194, Tehran, 1411713116 Iran; 2 School of Electrical and Computer Engineering, College of Engineering, University of Tehran, Tehran, 1439957131 Iran

**Keywords:** Metamaterials, Electrical and electronic engineering, Optoelectronic devices and components

## Abstract

We propose a metasurface THz modulator based on split-ring resonators (SRRs) formed by four interconnected horizontal Si–Au Schottky diodes. The equivalent junction capacitance of each SRR in the proposed modulator is much smaller than that of the previously reported metasurface counterparts with vertical Schottky junctions, leading to a higher modulation speed. To modulate a THz incident signal by the proposed metasurface, we vary the bias voltage externally applied to the Schottky junctions. Applying a reverse bias of *V*_A_ =  − 5 V to the Au gate, two LC resonances at 0.48 THz, and 0.95 THz are excited in the metasurface. Switching the applied voltage to *V*_A_ =  + 0.49 V, we diminish the oscillator strengths of the LC resonances, creating one dipole resonance at 0.73 THz in the transmission spectrum of the metasurface modulator. The modulation depths at these resonances are more than 45%, reaching 87% at 0.95 THz. The phase modulation for this THz modulator is about 1.12 rad at 0.86 THz. Furthermore, due to the particular design of the meta-atoms, the modulation speed of this device is estimated up to approximately several hundred GHz, which makes this device an appropriate candidate for high-speed applications in wireless communications systems based on external modulators.

## Introduction

The terahertz (THz) frequency range, due to its desirable features such as photon energy being several orders of magnitude less than the energy level of ionizing photons, terabit per second (Tbps) data rate, higher link directionality, lower vulnerability to eavesdropping, and less vulnerability to undesirable weather conditions, has attracted ample attention for application in next-generation ultra-fast wireless communication networks^1–4^. However, the weak response of many natural materials to THz radiation has posed many challenges to technological advancements in this area^5,6^. Nonetheless, the exceptional features of some metamaterials and their 2D counterparts (metasurfaces) have overwhelmed the challenges. A metasurface is a periodic array of meta-atoms (metal/dielectric elements of subwavelength dimensions). It is designed to control the incident wave amplitude, phase, or polarization state, desirably^7–10^. To date, researchers proposed a variety of THz devices, based on metamaterials and metasurfaces—e.g., lenses^11,12^, polarization convertors^13–16^, perfect absorbers^17–19^, waveplates^20^, and beam deflectors^21^. In these meta-devices, unlike in the conventional diffractive devices, what causes the incident wave phase and polarization to change, is not the wave propagation along and across the device. These changes rather happen abruptly and significantly over a thickness of only a fraction of the wavelength by the subwavelength scatterers^22^. Future systems are moving towards being intelligent and adaptive to their environment. Besides, nowadays, minds are thinking of the design of metasurfaces which can integrate several applications into a single ultra-thin device. However, many of the proposed metasurfaces are composed of passive elements, making the active tuning of the output electromagnetic properties of these devices impossible. Researchers have proposed various approaches to overwhelm this shortcoming for active and real-time control of the metasurfaces operations. Some of the latter methods work by changing the meta-atoms configuration or electromagnetic coupling between adjacent resonators, realized by MEMS technology^23,24^ or flexible substrates^25^. In other approaches, researchers used tunable materials like VO_2_^26,27^, SrTiO_3_^28^, Si^29,30^, perovskite^31^, or graphene^18,32,33^ in the unit cell structure. One can modulate the metasurface output response by modifying the properties of the tunable materials by a particular external stimulus like heat, light, or an electrical voltage. Despite their high modulation depth, the thermally tuned devices, among these methods, do not possess considerable modulation speed. Mechanical control suffers from the depreciation of the components. Though the optical technique yields the fastest modulation, among all other methods, it requires an expensive input optical source and light pump equipment, having its difficulties. Amongst the named modulation methods to tune a metasurface, the electrical approach, having appropriate modulation speed and bandwidth, large dynamic range, and being CMOS compatible is advantageous. An example of the electrically tuned THz metasurface is the one proposed in^34^. There, they modulated the incident THz waves by changing the bias applied to the Schottky junctions formed between the metasurface and the doped semiconductor substrate with a modulation speed in the kHz range. This work became the prelude to designing a doped semiconductor-metasurface composite tunable THz devices^21,35–39^, which at best have provided a modulation speed of several MHz. By hybridizing THz metasurfaces with pseudomorphic high electron mobility transistors (pHEMTs)^40–46^, a different type of electrically tuned THz modulators has emerged, recently. Although through this approach, the THz wave modulation is based on the changes in the carrier concentration of nm-thick two-dimensional electron gas (2DEG) layers by an electric bias voltage, achieving the GHz range modulation speed is possible. Such devices suffer from deficiencies, like low resonance strength and high insertion loss, and pose a tradeoff between modulation speed and depth improvement^45,46^. By placing PIN or varactor diodes in the gaps of meta-split-ring resonators (SRRs), one can convert the metasurface resonances in the microwave range of frequencies. This resonant mode conversion can include conversion from inductor-capacitor (LC) to LC or LC to a dipole.

In this paper, we show the procedure for designing a THz metasurface binary modulator consisting of SRRs whose gaps are filled by Si, forming Schottky diodes. This binary THz modulator modulates the co-polarized transmission of THz wave by converting two LC resonant modes to a dipole mode in the frequency range of 0.2–1 THz via switching the electric bias voltage from reverse to forward across the Schottky diodes formed between n-doped Si and metals forming the SRRs.

## Theory and design

**Figure 1:**
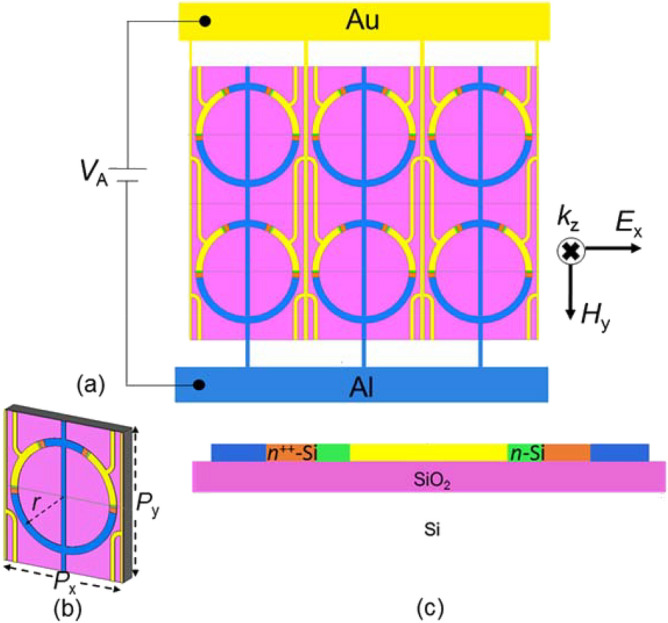
(**a**) A schematic top (*x–y*) view of the metasurface THz modulator with local and global interconnects and bias pads; (**b**) a 3D representation of the unit cell constituting the metasurface; (**c**) a 2D side (*r-z* plane) view of the unit cell, rotated to show the two adjacent (of four) Schottky diodes about a single Au gate symmetrically.

Figure 1a shows a schematic top (*x–y*) view of a portion of the proposed metasurface binary THz modulator, consisting of an array of 18 × 20 unit cells devised on a Si-on-insulator wafer. Figure 1b depicts a 3D schematic representation of the unit cell, composed of four parallel Au/n-Si Schottky diodes interconnected via two Au-gates and two standard low resistance tunnel ohmic contacts (Al/n^++^–Si). Figure 1c depicts a 2D cross sectional view of this unit cell, rotated in an *r-z* plane to show two adjacent Al/n^++^-Si/n-Si/Au Schottky diodes about a joint Au gate symmetrically.

As seen in Figure 1a, two types of metallic lines (Au and Al) interconnect the unit cells (in parallel) and provide the path to the external bias source. Table 1 lists the geometrical and physical parameters of the unit cell and its constituents. Electron-beam lithography (EBL) is the most flexible technique for direct printing of the sub-micrometer feature sizes (Table 1) with tolerances that has insignificant effects on the device characteristics^47–49^.

**Table 1:** Geometrical and physical parameters of the modulator unit cell and its constituents.

Symbol	Definition	Size	Dimension
*P*_*x*_	Unit cell period in the *x*-direction	85	µm
*P*_*y*_	Unit cell period in the *y*-direction	100	µm
*h*	SRR thickness	500	nm
*N*_D1_	Dopant concentration in n-Si	3 × 10^17^	cm^−3^
*N*_D2_	Dopant concentration in n^++^-Si	10^20^	cm^−3^
*N*_C_	Effective density of states in the Si conduction band	3 × 10^19^	cm^−3^
*l*_Au_	The average length of each Au arc in the SRR	40.5	μm
*l*_Al1_	The average length of the shorter Al arc in the SRR	27.882	μm
*l*_Al2_	The average length of the longer Al arc in the SRR	109.215	μm
*l*_Si_	The average length of each Si arc in the SRR	1.239	μm
*l*_n-Si_	The average length of the n-Si arc in the SRR	0.167	μm
*r*	SRR average radius	35.5	μm
*w*_R_	SRR width	5	μm
*w*_i_	Interconnect width	3	μm
*t*	The thickness of the SiO_2_ layer	1	μm
*m*_e_*	Conductivity effective mass in Si	2.46 × 10^−31^	kg
*ε*_0_	The free space permittivity	8.85 × 10^−12^	F/m
*ε*_r1(2)_	The relative permittivity of SiO_2_(Si)	3.9 (11.9)	–
*Φ*_S_	The n-Si workfunction	4.18	eV
*Φ*_Au_	The Au workfunction	4.7	eV
*Φ*_B_	The Schottky barrier height at n-Si/Au interface	0.65	eV
*Φ*_bi_	The Schottky diode built-in potential	0.52	eV
*σ*_Al_	Conductivity of Al	3.56 × 10^7^	S/m
*σ*_Au_	Conductivity of Au	4.56 × 10^7^	S/m
*µ*_e1_	Electron mobility in n- Si	474	cm^2^/V∙s
*µ*_e2_	Electron mobility in n^++^- Si	73	cm^2^/V∙s

**Figure 2:**
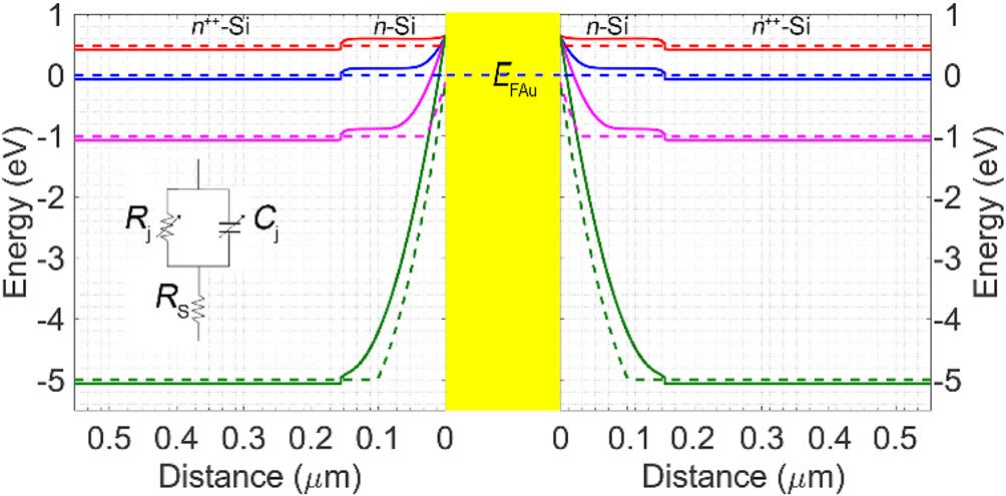
Energy band diagrams of the two adjacent Schottky diodes of Fig. 1c at thermal equilibrium (solid blue), the forward bias of *V*_A_ =  + 0.49 V (solid red), and the reverse biases of *V*_A_ =  − 1 V (magenta) and *V*_A_ =  − 5 V (solid green). The dashed curves represent the corresponding quasi-Fermi levels. The inset shows the equivalent circuit of a Schottky diode.

Solving the well-known Poisson equation by a finite element method (FEM), considering the parameters given in Table 1, and the Joyce-Dixon approximation^50^

1 $$\frac{{E_{F} - E_{C} }}{{k_{{\text{B}}} T}} \approx \ln \frac{{N_{D} }}{{N_{C} }} + \frac{1}{\sqrt 8 }\frac{{N_{D} }}{{N_{C} }},$$

we have calculated the energy band diagram across the Si layer in each Schottky junction, to gain an insight into the device electrostatics. Here, *k*_B,_ *T*, *E*_C_, and *E*_*F*_ are the Boltzmann constant, temperature, the conduction energy level, and the Fermi level. The solid curves in Fig. 2 show the band diagrams in two Si regions adjacent to a single Au gate, in thermal equilibrium (blue), forward bias of *V*_FB_ = (*Φ*_bi_ − *k*_B_*T* )/*q*≈ 0.49 V (red), and two reverse biases of *V*_R_ = 1 (magenta) and 5 V (green). The dashed curves depict the variation of quasi-Fermi levels across both junctions. In these calculations, we assumed an ideal Schottky interface. Because what influences our design significantly is the conceptual operation of each Schottky diode rather than the diode ideality factors^51^.

To explain the modulation mechanism of the transmission of an incident THz wave through the structure, first, we consider the small signal circuit model of a Schottky diode, as depicted in the inset of Fig. 2. The elements *R*_S_, *R*_j_, and *C*_*j*_ represent the diode series resistance, the variable junction resistance, and capacitance. One can calculate the junction capacitance with high accuracy for the applied voltages of *V*_*A*_ < (*Φ*_bi_ − 6*k*_B_*T*)/*q* using^52,53^:

2 $$C_{j} \approx A\sqrt {\frac{{\varepsilon_{0} \varepsilon_{{{\text{r2}}}} qN_{D} }}{{2\left( {{{\left( {\Phi_{bi} - k_{{\text{B}}} T} \right)} \mathord{\left/ {\vphantom {{\left( {\Phi_{bi} - kT} \right)} q}} \right. \kern-\nulldelimiterspace} q} - V_{A} } \right)}}} .$$

For *V*_A _= + 0.49 V, however, the effect of *C*_*j*_ can be ignored. Consider illuminating the proposed metasurface by a small signal THz electromagnetic wave. The in-plane component of the THz field can move carriers in the conductive regions of the SRR. The electric field component normal to the sidewalls of the depletion regions in the Schottky diodes (i.e., the SRR gaps) separates the positive and negative charges, accumulating them on the opposite sidewalls of the SRR gaps. In other words, the THz signal charges/discharges the capacitive elements. These bias-dependent capacitive elements that are almost frequency independent with the adjacent inductive metal parts induce LC resonance in each SRR. In other words, the THz small signal amplitude (i.e., << |*V*_A_|) causes an insignificant variation in the depletion layer width. Besides, the semiconductor dielectric relaxation time determines the time response of the majority carriers in the Schottky diodes (i.e., in the order of ps)^51^. As the solid green curves in Fig. 2 demonstrate, the n-Si regions of the Schottky diodes at *V*_R_ = 5 V are depleted almost entirely from the electrons, maximizing the effect of *C*_*j*_ in the LC resonance. Our simulations show that the maximum electric field within the junction at *V*_R_ = 5 V is less than the electrostatic strength (breakdown field) for the n-Si in an abrupt junction^54^. The solid red curves in Fig. 2 show as *V*_F_ →(Φ_bi_−*k*_*B*_*T)/q,* the depletion region diminishes, making the effect of the capacitive element ignorable in the LC resonance due to the dominance of the conduction current to the displacement current^51,53^. In other words, at this particular forward bias, the LC resonance modes are dampened, and only an electric dipole resonance is excited in the resonators. For 0 < *V*_A _< +0.49 V, the conductive path is not good enough to provide the desired dipole resonance. The conversion from LC resonance in the reverse bias to the dipole resonance in the forward bias is the basis for the modulation of the incident THz wave.

The small signal junction resistance (*R*_*j*_ in the inset of Fig. 2) is^55,56^:

3 $$R_{j} = \frac{\eta k_{{\text{B}}} T}{{q\left( {I + I_{S} } \right)}},{\text{with }}I = I_{S} \exp \left[ {\left( {{{qV_{A} } \mathord{\left/ {\vphantom {{qV_{A} } {kT}}} \right. \kern-\nulldelimiterspace} {k_{{\text{B}}} T}}} \right) - 1} \right]{\text{ and }}I_{S} = AA^{*} T^{2} \exp \left( {{{ - q\Phi_{B} } \mathord{\left/ {\vphantom {{ - q\Phi_{B} } {kT}}} \right. \kern-\nulldelimiterspace} {k_{{\text{B}}} T}}} \right),$$

where *η≈* 1.66*, A* = *h* × *w*_R_, and A^*^ = 246 A∙(K cm)^−2^^57^ are the ideality factor, cross-sectional area, and the effective Richardson constant for the electrons in Si Schottky diodes. Note that we adopted the value of *η* from ^58^ to be realistic.

The series resistance *R*_S_ is the sum of the resistances originating from the ohmic contact (Al/n^++^–Si) and the quasi-neutral region of the n-Si under the given bias. One can readily ignore the part of the series resistance related to the Al/n^++^-Si due to the heavy doping (10^20^ cm^−3^) of the n^++^-region. Moreover, for calculating the portion of *R*_*S*_ related to the undepleted Si-region in the structure, one can use the real part of the series impedance proposed by^59,60^, taking the high-frequency destructive effects of the charge carrier inertia, dielectric relaxation, and the skin effect into account. Although the method was originally prescribed for Schottky diodes with circular cross-sections, it can be used to estimate the series impedances of Schottky diodes with rectangular cross-sections^61^.

## Simulation results

**Figure 3:**
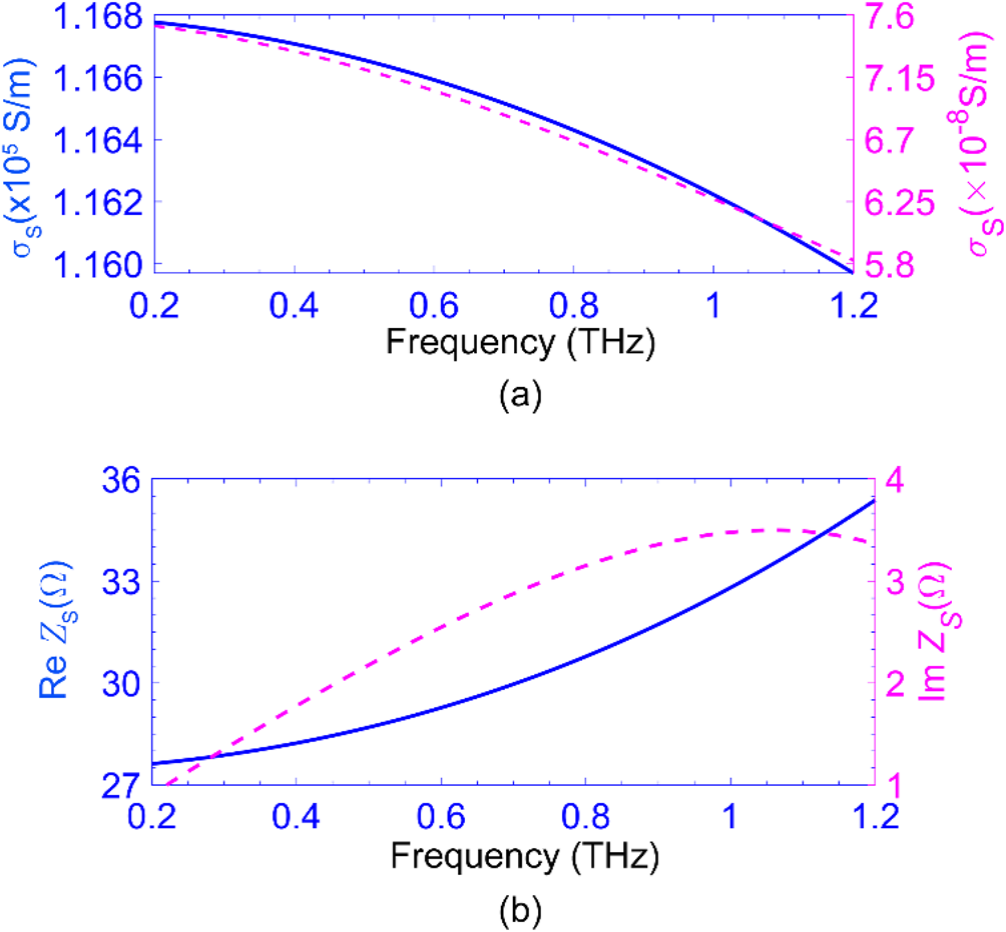
The Drude conductivity σ_*S*_ versus frequency for n^++^-Si (blue solid) and the entire depleted n-region at *V*_R_ = 5 V (magenta dashed); (b) Re *Z*_*S*_ (solid blue) and Im *Z*_*S*_ (dashed magenta) for undepleted n-region at *V*_F_ = 0.49 V.

To portray the modulation of the incident THz wave by the proposed metasurface, we performed a 3D fullwave electromagnetic simulation of the unit cell of Fig 1b, employing the finite element method (FEM). The application of periodic boundary conditions in the *x* and *y* directions portrays the metasurface 2D periodicity. For the accuracy of the simulation results, what matters here is the accurate modeling of the reverse- and forward-biased Schottky diodes in the electromagnetic simulation. As shown in Fig. 1a, the electric field of the incident THz wave normally impinging upon the metasurface modulator is along the *x*-axis (*E*_*x*_) so that it can excite the typical LC resonance in each SRR for *V*_R_ = 5V. In this situation, the electric field component perpendicular to the sidewalls of the SRR gaps induces a circular electric current in the metallic ring of the SRR. Although the strongest magnetic resonance of the SRR is inactive because *H*_*z*_= 0, the induced electric current produces a magnetic dipole normal to the SRR plane. These resonances are known as electric-generated magnetic resonances^62,63^. Considering an infinite array here, there is no out-of-plane radiation for these magnetic dipoles under normal incidence. Yet, the subwavelength periodicity of the proposed structure makes the total in-plane radiation insignificant. Besides, the electric dipoles induced in the Schottky junctions depletion regions radiate in the normal direction, resulting in co-polarized transmission and reflection. In addition to this magnetic resonance, other resonances may occur at higher frequencies within the propagating zero-order Floquet harmonic zone, attributed to the higher-order modes of the current distribution ^64^. It is noteworthy that a y-polarized incident wave can not excite any LC resonance and only an electric dipolar resonance takes place in each SRR. Moreover, we used the Drude model to describe the conductivity (σ_S_) and relative permittivity (ε_S_) of each Si segment in the Schottky diode in the THz range^40,42,65^:

4a $$\sigma_{S} = \sigma_{0} \left( {\frac{1}{{1 + j\left( {{\omega \mathord{\left/ {\vphantom {\omega {\omega_{s} }}} \right. \kern-\nulldelimiterspace} {\omega_{s} }}} \right)}}} \right)$$

and

4b $$\varepsilon_{S} = \varepsilon_{r2} - j{{\sigma_{S} } \mathord{\left/ {\vphantom {{\sigma_{S} } {\varepsilon_{0} \omega }}} \right. \kern-\nulldelimiterspace} {\varepsilon_{0} \omega }}$$

where *ω, σ*_0_ = *qμ*_*e*_*n*_*e*_, and *ω*_*s*_ = *q/m*_e_^*^µ_e_ represent the angular frequency, Si DC conductivity, the scattering frequency, or the damping rate. Notice, *n*_*e*_ (i.e.*,* the electron concentration) in the depleted section of the Schottky diode for *V*_R_ = 5 V is ~ *n*_e_ ≈ 1 × 10^7^ cm^−3^. For the electron mobility (*μ*_*e*_) and conductivity effective mass (*m*_e_^*^) in n- and n^++^-Si, see Table 1. Using the data for *ω*_s1(2)_ ≈ 89.1 (13.7) × 10^12^ rad∙s^−1^ for the n- and n^++^-Si and Eq. (4a), we have calculated σ_*S*_ versus the incident signal frequency. The blue solid and magenta dashed curves in Fig. 3a depict the real part of the σ_*S*_(*ω*) for the n^++^ (left axis) and entirely depleted n region (right axis) at the given reverse bias. The negligible electrical conductivity of the depleted n region, compared to that of the highly conductive n^++^-Si region, reveals that this region behaves like a quasi-insulator.

The Schottky diodes in the forward bias (*V*_F_ = 0.49 V) become conductive. Thus, unlike in the reverse case, we should take the effect of the junction resistance into account, ignoring the impact of the junction capacitance, as stated in the previous section. In this case, we should model the Schottky diodes by the lumped model in the simulation, employing the series impedance of each n-Si region with the assumption of no depletion ^60^:

5 $$Z_{S} = \frac{{l_{n - Si} }}{{\sigma_{0} A}}\left\{ {\frac{1}{{1 + j\left( {{\omega \mathord{\left/ {\vphantom {\omega {\omega_{S} }}} \right. \kern-\nulldelimiterspace} {\omega_{S} }}} \right)}} + j\left( {{\omega \mathord{\left/ {\vphantom {\omega {\omega_{d} }}} \right. \kern-\nulldelimiterspace} {\omega_{d} }}} \right)} \right\}^{ - 1}$$

where *ω*_*d*_ = *σ*_0_/*ε*_0_*ε*_r2_ represents the dielectric relaxation frequency in Si. By this description, the destructive effects associated with the charge carrier inertia and displacement current that increase the series impedance of the Schottky diodes are considered in the simulation ^59,60^.

In the forward bias, we replace the n-Si region in each Schottky diode with the impedance of the lumped element,—i.e., *Z*_S_ +*R*_*j*_. The solid blue line and the magenta dashes in Fig. 3b represent the Re *Z*_*S*_ (left axis) and Im Z_S_ (right axis). This figure shows Im *Z*_*S*_ ≪Re (*Z*_S_)+*R*_*j*_. Hence, the lumped element can be considered purely resistive—i.e., Re (*Z*_S_)+*R*_*j*_. Here too, the n^++^-Si is modeled by Eq. (4).

**Figure 4:**
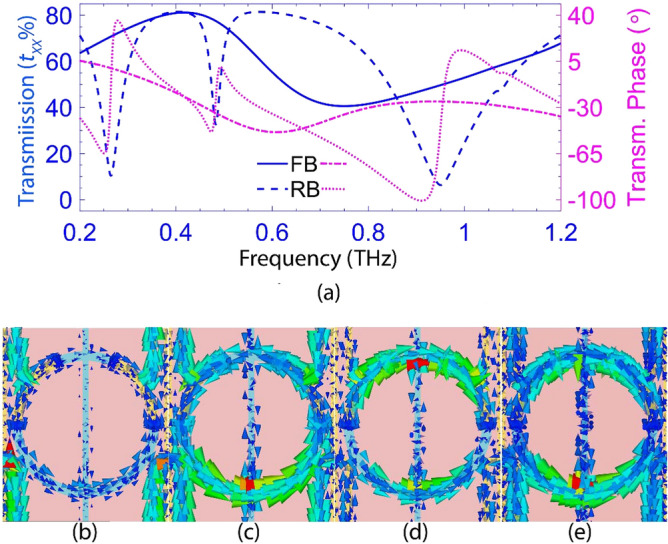
(**a**) The co-polarized transmission amplitude spectra at the forward (solid blue) and reverse (dashed blue) and phase spectra at the forward (magenta dotted-dashes) and reverse (magenta dots), (**b**)–(**d**) the surface current distributions at the dipole resonance of 0.26 THz and LC resonance frequencies of 0.48 and 0.95 THz, obtained in the reverse bias, (**e**) the surface current distributions at the dipole resonance frequency of 0.74 THz obtained at the forward bias.

To characterize the modulator, we evaluate the spectrum of the co-polarized transmission coefficient, defined as the ratio of the x-component of the output electric field amplitude to its input counterpart (i.e., *t*_*xx*_ = |*E*_*x-*out_|/|*E*_*x*-in_|). It is noteworthy that the cross-polarized components are negligible due to the symmetry concerning the y-axis. The blue dashed (solid) line in Fig. 4a represents the calculated spectrum at the reverse (forward) bias. The dashed curve shows, at *V*_R_=5 V, there are three significant resonances manifested by the transmission minima at 0.26, 0.48, and 0.95 THz. Fig. 4b–d demonstrate the distributions of the surface currents at these resonances. The resonance at 0.26 THz is a dipole resonance, in which the surface current crowds on a path consisting of the local interconnects of four adjacent unit cells (Fig. 4b). To embed the bias lines on the planar structure, we have designed the local and global interconnects in this fashion for two reasons. First, the global interconnects are aligned in the y-direction to have minimal effect on the electromagnetic device response. Second, the local interconnects lengths are chosen so that the corresponding resonance (at 0.26 THz) is isolated from the other two resonances related to the SRR. The typical LC resonance of the SRR occurs at 0.48 THz, and the corresponding surface current distribution in Fig. 4c shows that a circulating current flows with only one maximum point. The effective perimeter of the SRR in this LC resonance is *l*_eff_ ≈ *λ*_eff_/2, in which *λ*_eff_ is the corresponding resonance wavelength. Besides, at this resonance, known as magnetic resonance, a strong electric field is confined in the depletion regions of the Schottky junctions. The third resonance in the transmission at 0.95 THz also takes place in the SRR. This resonance is not a high-order Floquet harmonic because its frequency does not alter with the periodicity changing (According to (6)). So, it is another SRR LC resonance within the zero-order Floquet zone. According to Fig. 4d, the SRR resonates in this frequency due to the third-order mode of the current distribution with three maximum points and the estimated resonance wavelength of *λ*_eff_ ≈ 2 *l*_eff_ /3. The excitation of the LC resonances and the corresponding frequencies depend on the number and position of the Schottky junctions in the ring resonator. Because the orientation of the depletion layers relative to the incident electric field polarization changes the capacitance value, shifting the LCs’ resonance frequencies. The larger the number of the Schottky junctions, the smaller the total inductance (*L*) originating from the conductive regions of each SRR—i.e., Au, Al, and the n++-Si. Furthermore, placing the Schottky junctions at positions where the surface current peaks cause the LC resonances to dampen.

Moreover, the solid blue curve in Fig. 4a shows the co-polarized transmission through the metasurface, at the forward bias of *V*_F_ =0.49 V, exhibits a wide dipole resonance at ~0.74 THz, while the LC resonant modes are damped. In other words, the depletion region in each n-Si segment has disappeared, short-circuiting the Au and n-Si contacts in each SRR. Hence, the dipole-like resonance in each SRR enjoys a surface current distribution shown in Fig. 4e. Also, the dipole resonance at 0.26 THz disappears because the conducting Schottky contacts make the current path longer, shifting the corresponding dipole resonance to a much lower frequency. This shift is the cause of the transmission reduction towards lower frequencies observed in the solid blue curve. This active control of the metasurface resonant modes and the conversion of LC resonances to a dipole resonance by altering the electrical bias of the Schottky junctions from reverse to forward and vice versa results in an efficient modulation of incident THz waves at these resonance frequencies. The corresponding modulation depths—i.e., MD = (*t*_max_−*t*_min_)/*t*_max_ with min and max indicating the minimum and maximum values—are given in Table 2.

**Table 2:** The Modulation depths of the LC and dipole resonances at their corresponding frequencies.

	Resonance type			
	Reverse bias			Forward bias dipole
	Dipole	LC		
Frequency	0.26 THz	0.48 THz	0.95 THz	0.74 THz
MD	85%	58%	87%	45%

According to the Kramers-Kroning relation, wherever the amplitude modulation is maximum, the phase modulation is minimum, and vice versa^44^. Therefore, we investigated the phase shifting of the THz radiation by the modulator. The magenta dots and dots-dashes in Fig. 4a depict the spectra of the co-polarized transmission phase for the metasurface modulator at the given reverse and forward biases. Each shows a phase jump about the corresponding resonance, regardless of its type (LC or dipole), whose height depends on the resonance strength. From Fig. 4a, one can see that at frequencies for which the change in the transmission amplitude is the largest, the phase modulation is the smallest. Besides, the most significant phase jump of ~114° in the LC resonance occurs around 0.95THz. We also achieved the highest phase modulation of ~64.2° appearing at ~ 0.86 THz with less than a 5.5 V change in the applied bias. It is twice that of the Schottky-based modulator reported in^36^, obtained with about three times larger bias change (16 V). Hence, we may use this phase modulator in designing THz phase shifters.

For validation of the results just we have reported, we may use an equivalent circuit model for each bias case. Theoretically, one can use an appropriate lumped circuit model to analyze a periodic frequency selective surfaces (FSS) if the operating frequency (*f*_0_) is much lower than the first higher-order mode (grating lobe) cut-off frequency^64,66^,

6 $$f_{{\text{C}}}^{m\;n} = \frac{c}{{\sqrt {\varepsilon_{r} } }} \times \sqrt {\left( {\frac{m\,}{{P_{x} }}} \right)^{2} + \left( {\frac{n\,}{{P_{y} }}} \right)^{2} } ,$$

where *ε*_r_ is the dielectric constant of the mediums surrounding the FSS, wherein *m* (*n*) = 0, ± 1, ± 2, …, corresponds to the order of the Floquet harmonics excited due to the FSS periodicity under normal incidence. Consider an electromagnetic wave of frequency *f*_0_ impinging normally upon an FSS like the proposed metasurfaces, satisfying the condition *f*_*c*_^*mn*^ ≫ *f*_0_ (Here, *m* and *n* indicate the order of the first higher-order mode). Hence, only its fundamental floquet harmonic propagates, and the higher-order modes evanesce.

**Figure 5:**
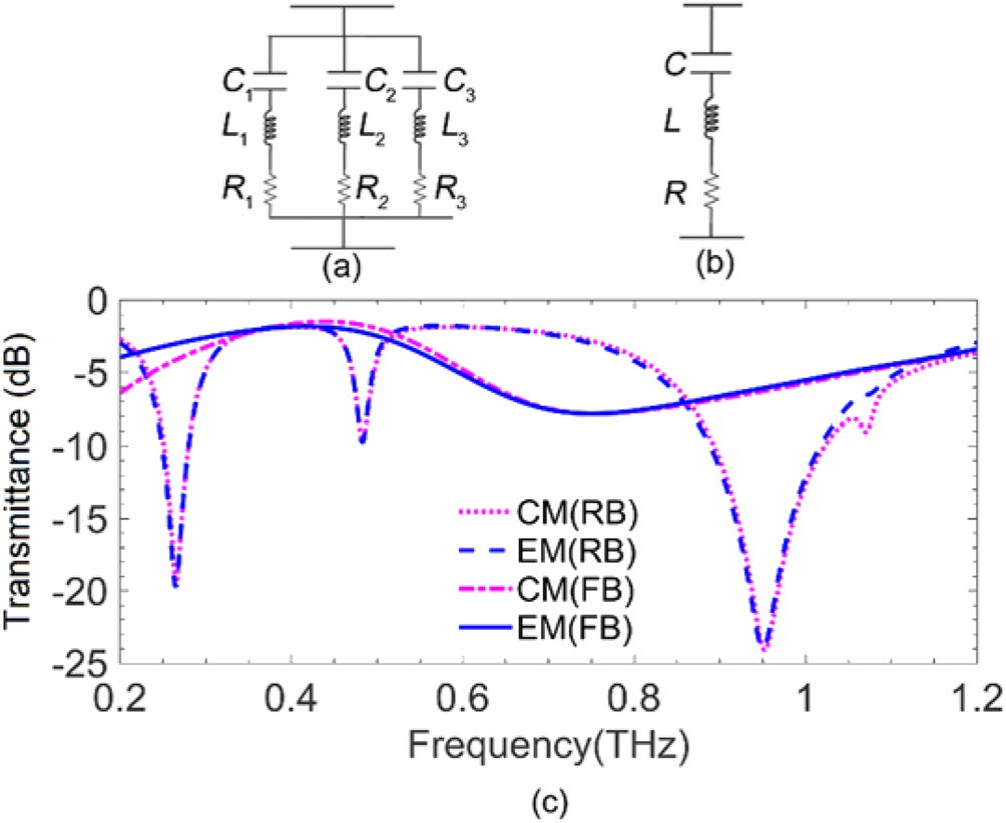
The equivalent circuit model of the metasurface for the (**a**) reverse and (**b**) forward bias of the Schottky, (**c**) The transmission spectra obtained from the equivalent circuit model (magenta dots and dots-dashes curve), and the full-wave simulation (blue dashed and solid curve) in the presence of the ohmic losses for reverse and forward bias cases.

Figure 5a and b illustrate the equivalent circuit model of the metasurface at the reverse bias and forward bias, respectively. Each branch in the reverse bias model—i.e., an inductor, a capacitor, and a resistor in series—represents one of the three resonances—i.e., one dipole resonance and two LC resonances—labeled with indices 1, 2, and 3. It is well-established to model the SRR with a series RLC lumped circuit model in which R, L, and C represent the loss in metallic and high-conductive regions, the inductance of the conductive ring, and the total capacitance of the gaps, respectively. Therefore, this model, obtained from the element geometry, can describe the physical behavior of the structure but only in the fundamental magnetic resonance of the SRR (Here at 0.48 THz). Yet, one can extend the circuit model, including the other resonance within the propagative zero-order Floquet zone originating from the third-order current distribution, by considering some extra lumped elements. However, no physical interpretation exists for the extended model^64^. On the other hand, the transmission phase (Fig. 4a) around the resonances reveals that the metasurface impedance is capacitive before each resonance, becoming inductive afterward, just like a series RLC circuit. We exploit the method described in^64,67^ for evaluating their quantities (See Supplementary for details).

The optimized quantities of the lumped elements of the equivalent circuit models are given in Table 3. The magenta dots and dots-dashes in Fig. 5c, depict the spectra for the transmittance (|*t*_CM_|^2^) in dB related to the equivalent circuit model in reverse and forward bias, using quantities in Table 3. The blue dashed curve and solid curve in Fig. 5c, characterize the transmittance (|*t*_EM_|^2^) in dB through the reverse- and forward-biased metasurface, obtained from the electromagnetic full-wave simulation. A quick comparison reveals excellent matches between the transmittance spectra obtained from both methods for either of the two biasing conditions almost over the entire range of frequency shown.

**Table 3:** Quantities of the lumped elements of the equivalent circuit models of Fig. 5a and b.

Bias	Lumped elements								
	Inductance (nH)			Capacitance (fF)			Resistance (Ω)		
Reverse	*L*_1_	*L*_2_	*L*_3_	*C*_1_	*C*_2_	*C*_3_	*R*_1_	*R*_2_	*R*_3_
	0.22	0.86	0.05	1.64	0.13	0.6	12	55	7
Forward	L	C	R						
	0.04	1.16	80						

The desired operation of the proposed metasurface binary modulator relies on the appropriate switching speed of the Schottky diodes between the On and Off states. The analytical approach to estimate the switching speed here is the use of the Schottky diodes cut-off frequency (i.e., *f*_*C*_ = 1/(2*πR*_*S*_*C*_*j*0_), ignoring the parasitic capacitances)^59,68^, indicating the upper limits of the diodes speed to change between the On and Off states. Notice, *C*_*j*0_ ≈ 5.63 fF (setting *V*_A_=0 in Eq. (2)) and *R*_S_ = Re [*Z*_S1_(*ω*) + *Z*_S2_(*ω*)], ignoring the skin effect due to the identical cross-sectional areas of the Schottky and ohmic contacts. Here, *Z*_S1(2)_(*ω*) follows Eq. (5) for the undepleted region of n (n^++^)-Si*.* In the calculation of *f*_*C*_, we considered the worst case, giving the smallest switching speed of the diodes (i.e., for the largest possible junction capacitance and series resistance for the given physical and geometrical parameters). Note that to obtain the metasurface cut-off frequency, we should consider its effective capacitance and total series resistance, knowing the *N* unit cells in the array and the four Schottky junctions in each unit cell are connected in parallel. Hence, the product of the array total series resistance and effective capacitance equals *R*_*S*_*C*_*j*0_ for an individual diode. In other words, the cut-off frequency of a single Schottky junction represents the upper limit of the Schottky diodes array switching speed between the On and Off states, resulting in *f*_*C*_ = 0.82 THz.

## Conclusion

We proposed a single-layer electrically tunable metasurface binary THz modulator in this paper. The metasurface unit cell is a split ring resonator composed of two pairs of common-gate Au/n-Si/n^++^-Si/Al Schottky diodes, devised on a Si-on-insulator substrate. The desired modulation becomes possible when the Schottky diodes are switched between two particular forward and reverse biases—i.e., On and Off states. This switching function enables the conversion from dipole resonance to LC resonance and vice versa. The role of local and global metal interconnects (Au or Al) is crucial in this design. Their design has led to a dipole resonance at 0.26 THz that is isolated from the two LC resonances related to the SRRs—i.e., 0.48 and 0.95 THz—having minimal effect on the electromagnetic of the modulator response. The maximum and minimum modulation depths obtained for this binary amplitude modulator are 87% (at the LC resonance of 0.95 THz) and 45% (at the only dipole resonance of 0.74 THz). Besides, the achieved phase modulation is 1.12 rad at 0.86 THz. Also, the Schottky diodes array switching speed, ignoring the effects of other parasitic capacitances, is estimated to be 820 GHz. Taking parasitic capacitances into account, one can get a switching speed of about several tens of GHz at the expense of less than 5.5 V bias change. This speed is much faster than that of other Schottky diode-based modulators reported in the literature obtainable with higher voltage change. Such impressive modulation depths and speeds make the proposed THz modulator a potential choice for various THz applications such as THz communications and imaging.

## Supplementary Information

Supplementary Information.

## Data Availability

Data underlying the results presented in this paper are not publicly available at this time but may be obtained from the corresponding author upon reasonable request.
