# Supplementary material for: Binary THz modulator based on silicon Schottky-metasurface
Source: Sci Rep. 2022 Nov 7;12:18871. doi: 10.1038/s41598-022-23534-w (PMC9640677; doi:10.1038/s41598-022-23534-w)
Supplement: Supplementary file 1 — Supplementary Information. [file 41598_2022_23534_MOESM1_ESM.docx]

Supplementary:

Binary THz Modulator Based on **Sil**icon Schottky-Metasurface


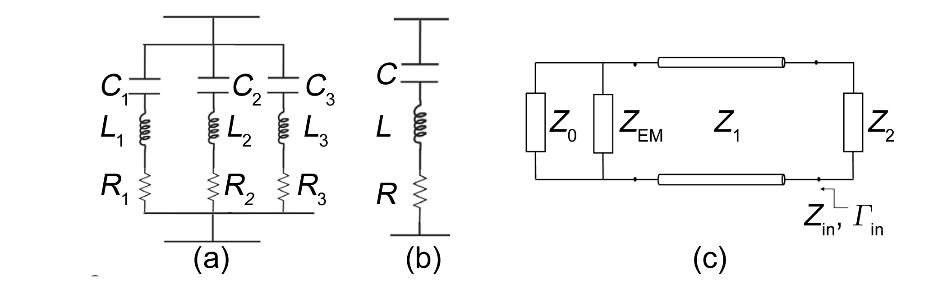


Figure S1. The equivalent circuit model of the metasurface for the (a) reverse and (b) forward bias of the Schottky, and (c) the transmission line model.

Here, we explain in detail the calculation of the circuit model lumped elements of the modulator in reverse and forward biases. Figures S1(a) and S1(b) illustrate the equivalent circuit model of each SRR in the metasurface at the reverse bias and forward bias, respectively. Each series resistor represents the loss attributed to the surface current path corresponding to the resonance. We obtain the inductance and capacitance quantities in the absence of loss for which the metasurface impedance is pure imaginary. With an initial guess for quantity L_1_, we minimize the Euclidean distance between the equivalent impedance of the metasurface circuit model,

$Z_{\text{CM(RB)}}=\frac{\left(1-\omega^{2}L_{1}C_{1}\right)\left(1-\omega^{2}L_{2}C_{2}\right)\left(1-\omega^{2}L_{3}C_{3}\right)}{j\omega\left[C_{3}\left(1-\omega^{2}L_{1}C_{1}\right)\left(1-\omega^{2}L_{2}C_{2}\right)+\left(1-\omega^{2}L_{3}C_{3}\right)\left(C_{1}+C_{2}-\omega^{2}C_{1}C_{2}\left(L_{1}+L_{2}\right)\right)\right]},$ (S1)

and calculated the impedance by using full-wave electromagnetic simulations (Z_EM_) in an iterative process. Equation (1) has three zeros at resonant frequencies of *ω_z_*_1_, *ω_z_*_2_, and *ω_z_*_3_, for which the metasurface impedance acts as a short circuit, minimizing the transmission coefficient. Thus, we have

$C_{1}=\frac{1}{L_{1}\omega_{z1}^{2}},\text{ }L_{2}=\frac{1}{C_{2}\omega_{z2}^{2}},\text{ }L_{3}=\frac{1}{C_{3}\omega_{z3}^{2}},$ (S2a)

$C_{2}=C_{1}\frac{\frac{\omega_{z1}^{2}\left(\omega_{z3}^{2}-\omega_{p3}^{2}\right)}{\omega_{z3}^{2}\left(\omega_{z1}^{2}-\omega_{p3}^{2}\right)}-\frac{\omega_{z1}^{2}\left(\omega_{z3}^{2}-\omega_{p2}^{2}\right)}{\omega_{z3}^{2}\left(\omega_{z1}^{2}-\omega_{p2}^{2}\right)}}{\frac{\omega_{z2}^{2}\left(\omega_{z3}^{2}-\omega_{p2}^{2}\right)}{\omega_{z3}^{2}\left(\omega_{z2}^{2}-\omega_{p2}^{2}\right)}-\frac{\omega_{z2}^{2}\left(\omega_{z3}^{2}-\omega_{p3}^{2}\right)}{\omega_{z3}^{2}\left(\omega_{z2}^{2}-\omega_{p3}^{2}\right)}},$ (S2b)

and

$C_{3}=-C_{1}\frac{\left(1-\omega_{p2}^{2}/\omega_{z3}^{2}\right)}{\left(1-\omega_{p2}^{2}/\omega_{z1}^{2}\right)}-C_{2}\frac{\left(1-\omega_{p2}^{2}/\omega_{z3}^{2}\right)}{\left(1-\omega_{p2}^{2}/\omega_{z2}^{2}\right)},$ (S2c)

wherein *ω_p_*_2_ and *ω_p_*_3_ represent two of the three frequencies, besides *ω_p_*_1_=0, for which the equivalent impedance has poles. In the forward bias, the device model consists of an inductor (*L*), a capacitor (*C*), and a resistor (*R*) connected in series (Figure S1(b)) because by switching the diodes to their conductive mode, we excite a dipole resonance in each resonator, as mentioned before. We exploit the same iterative method as for the reverse bias to calculate the inductor and capacitor quantities using an initial guess for *L*. In the absence of the resistive loss, the impedance of the metasurface related to the equivalent circuit model is

$Z_{\mathrm{CM} (\mathrm{FB})}=j\omega L+\frac{1}{j\omega C},$ (S3)

having one zero at the resonant frequency of *ω_z_*,

$C=1/L\omega_{z}^{2}.$ (S4)

We use the transmission line model (Figure S1(c)) for calculating Z_EM_ for the metasurface in reverse and forward bias cases. The element *Z*_1(2)_ = *ωµ*_0_ /*𝛽*_1(2)_ represents the characteristic impedance of the SiO_2_ (Si) layer below the metasurface, and *𝛽*_1(2)_=(*ω/c*)𝜀_r1(2)_^1/2^ is the related wave propagation constant in the propagation (z) direction within the layer. According to the classical transmission line theory, the relations for the input impedance (*Z*_in_) and the corresponding reflection coefficient (Γ_in_) extracted from the *S*_11_ parameter of the scattering matrix obtained from the full-wave simulations are:

$Z_{\text{in} \text{(EM)}}=Z_{1}\frac{Z_{L}+jZ_{1}\tan\left(\beta_{1}t\right)}{Z_{1}+jZ_{L}\tan\left(\beta_{1}t\right)}$ (S5)

and

$\Gamma_{\text{in}}=\frac{Z_{in}-Z_{2}}{Z_{in}+Z_{2}}.$ (S6)

The load impedance in (5) is

$Z_{L}=\frac{Z_{0}Z_{\mathrm{EM}}}{Z_{0}+Z_{\text{EM}}},$ (S7)

wherein *Z*_0_ ≈ 377 Ω is the free space characteristic impedance, and *Z*_EM_ can be obtained via some algebraic manipulations of (5)-(7):

$Z_{\text{EM}}=\frac{-Z_{0}Z_{1}Z_{2}\left(1+\Gamma_{\text{in}}\right)+j\tan\left(\beta_{1}t\right)Z_{0}Z_{1}^{2}\left(1-\Gamma_{\text{in}}\right)}{Z_{0}Z_{1}\left(\Gamma_{\text{in}}-1\right)+Z_{1}Z_{2}\left(1+\Gamma_{\text{in}}\right)+j\tan\left(\beta_{1}t\right)Z_{1}^{2}\left(\Gamma_{\text{in}}-1\right)+j\tan\left(\beta_{1}t\right)Z_{0}Z_{2}\left(1+\Gamma_{\text{in}}\right)}.$ (S8)

The transmission coefficient amplitude associated with *Z*_EM_ is the S_21_ element of the scattering matrix obtained from the electromagnetic full-wave simulation.

Now, to calculate the transmission coefficient amplitude (*t*_CM_) related to the equivalent circuit model, through the reverse and forward-biased metasurface, we need to include the data for the ohmic loss, represented by the resistive elements shown in Figures S1(a) and S1(b). In doing so, we follow the transmission line model shown in Figure S1(c), wherein

$Z_{\text{in} \text{(CM)}}=\frac{AZ_{0}+B}{CZ_{0}+D}$ (S9)

is the corresponding input impedance obtained according to the transmission line theory, with A, B, C, and D being the elements of the transfer ABCD matrix,

$\left(\begin{matrix} A & B \\ C & D \end{matrix}\right)\equiv\mathbf{M}_{\mathrm{SiO}_{2}}\times\mathbf{M}_{\mathrm{Metas}},$ (S10a)

in which

$\mathbf{M}_{\mathrm{SiO}_{2}}=\left[\begin{matrix} \cos\left(\beta_{1}d_{1}\right) & jZ_{1}\sin\left(\beta_{1}d_{1}\right) \\ jZ_{1}^{-1}\sin\left(\beta_{1}d_{1}\right) & \cos\left(\beta_{1}d_{1}\right) \end{matrix}\right] ,$ (S10b)

representing the SiO_2_ layer and

$\mathbf{M}_{\mathrm{M}\text{etas}}=\left[\begin{matrix} 1 & 0 \\ Z_{\mathrm{CM}-\text{L}}^{-1} & 0 \end{matrix}\right]$ (S10c)

that portrays the metasurface. The parameter *Z*_CM-L_ in (10c) is the modified impedance of the metasurface corresponding to the equivalent circuit model, taking the effect of resistive elements (Figures S1(a) and S1(b)) into account.

Substituting (9) in (6) for *Z*_in_ and using the resulting Γ_in_ we can calculate the transmission coefficient related to the circuit model:

$t_{\text{CM}}=\sqrt{1-\left|\Gamma_{\text{in}}\right|^{2}-\left|a\right|^{2}},$ (S11)

In (11), the absorption coefficient, *a*, originates from the ohmic losses acquired from the full-wave simulation. In this step, we have minimized the difference between *t*_CM_ and *t*_EM_ spectra by tuning the resistive elements of the models in Figures S1(a) and S1(b) in the reverse and forward biases.
